# Michael Barrett, M. A. M. D.

**Published:** 1887-07

**Authors:** 


### OBITUARY.

### MICHAEL BARRETT, M. A. M. D.

Dr. Barrett, so well known to many readers of this Journal, was born in London, England, in 1816, consequently, had attained the ripe age of 71 years at his death, which took place in the spring of 1887.

In regard to his early life nothing is known that would indicate he had any special fondness for those departments of science in which he so excelled, after his powers had fully developed. The son of a barrister, he seemed to turn with distaste from the law office, and delighted himself with the ships which crowded the river Thames. So fond was he of sailing, that he was sent out as a sailor, in which capacity he spent two years of his life. His father, however, determined to remove to Canada, and the subject of this sketch accompanied him as far as Toronto. Here, however, a separation soon took place, the father taking his family to Natchez, Miss., where they ultimately settled. Young Barrett remained in Canada. Shortly after he had arrived here, the struggle known as the rebellion of 1837 took place. Michael Barrett joined the forces which were fighting against the insurgents, but was not, as far as known, called to take any active part in the contest.

During the year 1837 or 1838 his marriage took place—a marriage which proved an eminently happy one.

In 1845 he became connected with Upper Canada College. He had not, at this time studied medicine, or taken any degree in arts. In 1849 he became Master of Arts, graduating from Toronto University. During the ensuing years he studied medicine, and such remarkable ability did he display as a student, that, on the completion of his course in 1852, he was appointed Lecturer in Chemistry in Toronto School of Medicine. He was now 36 years of age, and after holding the chair of chemistry a few years, he was appointed to that of Physiology. This appointment he held with distinguished success up till the day of his death. In 1870 he was selected as lecturer on Animal Physiology in the Ontario Veterinary College; this chair he also held at the moment of his decease.

In 1880 and '81 a severe illness so prostrated Dr. Barrett that he was unable to fulfil his arduous duties. At this time he was holding positions in Upper Canada College, Toronto School of Medicine and the Ontario Veterinary College. He resigned his chair in Upper Canada College, but in the other two institutions, so great was the anxiety to retain his services, that the place was temporarily filled, in the hope he might be able to resume the duties after a period of rest. Fortunately, this expectation was realized, and the following session to the delight of the students, he appeared in his accustomed place. He had now reached the ripe age of 66, an age at which most men seek to retire. But Dr. Barrett was an enthusiast in medical education, and, seeing the need in Toronto of a Woman's Medical College, he, in 1883, suceeded in establishing the present prosperous institution of that name, holding the position in it of Dean of the Faculty and Professor of Physiology.

With the buoyancy of youth did this distinguished physiologist fulfil his duties which were so heavy that they would have overtaxed the energy of many a young man—till the year before his death. In 1886 Mrs. Barrett was taken from him, and he never recovered from this crushing blow. The partner of his joys and sorrows for close on fifty years, he had so loved her that it seemed impossible to live without her, But, although the joy had gone out of his life, he bravely bore up to the last. On the Friday before his death, he lectured at Toronto School of Medicine, and at the Ladies' College. On Saturday morning he lectured at the Veterinary College. At one o'clock he took lunch with his family, at half-past two he was found dead in his drawing-room.

Dr. Barrett was connected with Upper Canada College for more than thirty years, with the Toronto School of Medicine for thirty-five years, with the Veterinary College sixteen, and with the Womans' Medical College for four years. Most of the legal and medical men of Canada, and very many of those of the United States, were thus under his professional care. But it is especially of his connection with the Veterinary College that we wish to speak. Out of the one thousand students who have attended this college up to the present time, more than eight hundred have come directly under Dr. Barrett's instruction, as Professor of Physiology. The influence he has thus had it is impossible to estimate. While a student in the college in 1870–71 the present writer first listened to his lectures. The charm of these lectures made an ineffaceable impression on the mind. Afterward associated with him on the teaching staff, it was impossible not to admire him the more, as acquaintance ripened into friendship. As a lecturer, the eloquence and clearness of his style, together with his remarkable geniality of manner, made him the idol of the students of all the colleges. But especially did he appreciate, and was appreciated by the veterinary students. Often has he expressed the pleasure which he experienced in lecturing to them. And by many tangible proofs did the students show their affection for him. On the day of the funeral such a concourse of students as perhaps never before, in this city at least, showed their love for a deceased professor, sadly followed his remains to their resting place—thus honoring themselves as well as the well-loved memory of the lamented Dr. Michael Barrett.

